# Supplementary material for: Modulation of Macrophage Response by Copper and Magnesium Ions in Combination with Low Concentrations of Dexamethasone
Source: Biomedicines. 2022 Mar 24;10(4):764. doi: 10.3390/biomedicines10040764 (PMC9030383; doi:10.3390/biomedicines10040764)
Supplement: Supplementary file 1 [file biomedicines-10-00764-s001.zip › biomedicines-1612644-SM.pdf]

biomedicines

MDPI

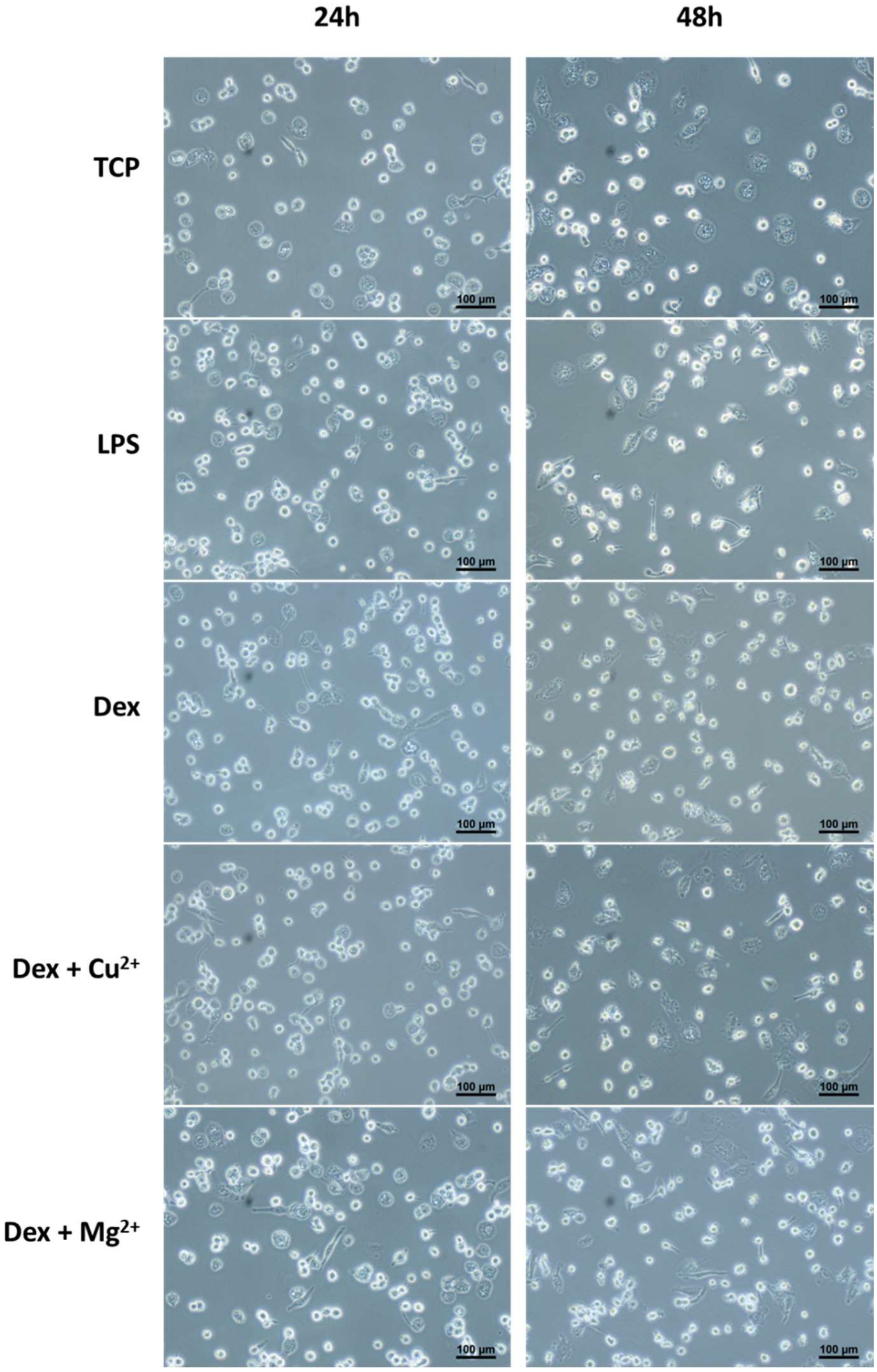

Figure S1. Effect of dex and the combination of dex with Cu^{2+} and Mg^{2+} on LPS-activated THP-1 macrophage cell line morphology. Scale bars = 100µm.

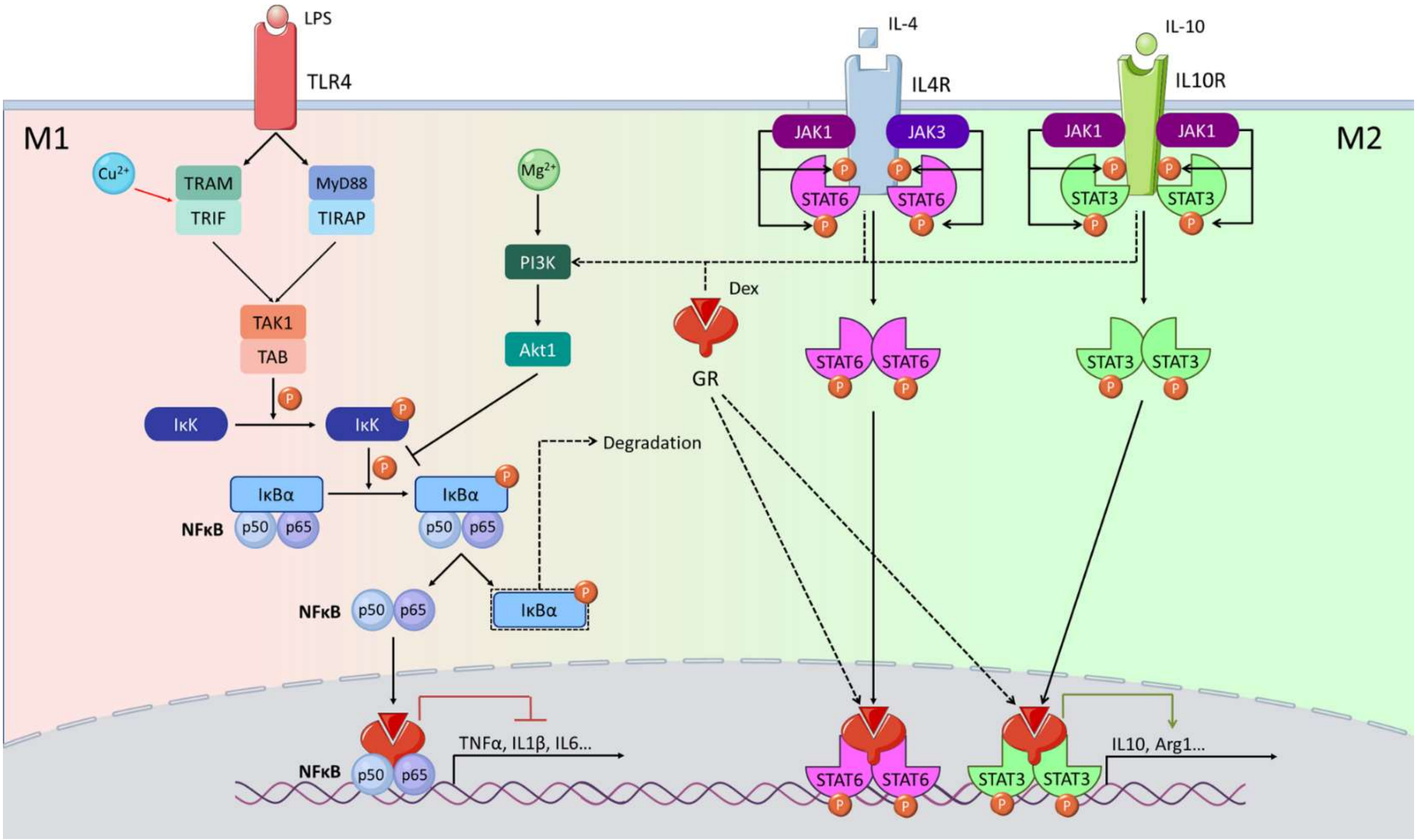

Figure S2. Schematic illustration of the signaling pathways involved in the M1 and M2 polarization of macrophages. Gene expression of M1 markers occurs mainly through NFκB pathway, while M2 marker expression is controlled by JAK-STAT and PI3K-Akt pathways. Cu^{2+} induced the activation of NFκB through a MyD88 independent pathway. Mg^{2+} promoted the activation of PI3K-Akt pathway, which inhibits the activation of NFκB pathway. The activation of the glucocorticoid receptor (GR) stimulates PI3K-Akt pathway and binds NFκB and STAT transcription factors either to act as a correpressor or as a coactivator, respectively.

Table S1. Statistical analysis of the results of TNF-*α* expression when THP-1 macrophages were stimulated with a combination of Cu^{2+} or Mg^{2+} with dex. Statistically significant differences were indicated with *($p < 0.05$).

	.	TCP	LPS	Dex	Cu	Mg	CuD	MgD
24h	TCP		*	*	*	ns	*	*
	LPS			*	*	*	*	*
	Dex				*	ns	ns	ns
	Cu					ns	*	ns
	Mg						*	ns
	CuD							ns
	MgD							
48h	TCP		*	ns	*	ns	*	ns
	LPS			*	*	*	*	*
	Dex				*	ns	*	ns
	Cu					ns	*	*
	Mg						*	ns
	CuD							*
	MgD							

Table S2. Statistical analysis of the results of IL-1β expression when THP-1 macrophages were stimulated with a combination of Cu^{2+} or Mg^{2+} with dex. Statistically significant differences were indicated with *($p < 0.05$).

		TCP	LPS	Dex	Cu	Mg	CuD	MgD
24h	TCP		*	*	*	ns	*	ns
	LPS			*	*	*	*	*
	Dex				*	*	ns	ns
	Cu					ns	*	ns
	Mg						*	ns
	CuD							ns
	MgD							
48h	TCP		*	*	*	ns	*	*
	LPS			*	*	*	*	*
	Dex				ns	*	ns	ns
	Cu					*	*	ns
	Mg						*	*
	CuD							*
	MgD							

Table S3. Statistical analysis of the results of CCR7 expression when THP-1 macrophages were stimulated with a combination of Cu^{2+} or Mg^{2+} with dex. Statistically significant differences were indicated with *($p < 0.05$).

		TCP	LPS	Dex	Cu	Mg	CuD	MgD
24h	TCP		*	ns	ns	*	*	*
	LPS			*	*	*	*	*
	Dex				ns	*	ns	ns
	Cu					*	*	ns
	Mg						*	*
	CuD							ns
	MgD							
48h	TCP		*	*	*	*	*	*
	LPS			*	*	*	*	*
	Dex				ns	ns	*	ns
	Cu					ns	*	*
	Mg						*	*
	CuD							ns
	MgD							

Table S4. Statistical analysis of the results of IL-10 expression when THP-1 macrophages were stimulated with a combination of Cu^{2+} or Mg^{2+} with dex. Statistically significant differences were indicated with *($p < 0.05$).

		TCP	LPS	Dex	Cu	Mg	CuD	MgD
24h	TCP		*	ns	*	*	*	*
	LPS			*	ns	ns	ns	ns
	Dex				*	*	*	*
	Cu					*	*	ns
	Mg						ns	ns
	CuD							ns
	MgD							
48h	TCP		*	*	*	*	*	*
	LPS			ns	ns	ns	*	ns
	Dex				ns	ns	*	ns
	Cu					ns	ns	ns
	Mg						ns	ns
	CuD							ns
	MgD							

Table S5. Statistical analysis of the results of TGF-β expression when THP-1 macrophages were stimulated with a combination of Cu2+ or Mg2+ with dex. Statistically significant differences were indicated with *($p < 0.05$).

		TCP	LPS	Dex	Cu	Mg	CuD	MgD
24h	TCP		*	ns	ns	ns	*	ns
	LPS			*	*	ns	ns	ns
	Dex				ns	ns	*	ns
	Cu					ns	ns	ns
	Mg						ns	ns
	CuD							ns
	MgD							
48h	TCP		ns	*	*	*	*	*
	LPS			*	*	*	*	ns
	Dex				*	ns	*	*
	Cu					ns	ns	ns
	Mg						ns	ns
	CuD							*
	MgD							

Table S6. Statistical analysis of the results of CD206 expression when THP-1 macrophages were stimulated with a combination of Cu^{2+} or Mg^{2+} with dex. Statistically significant differences were indicated with *($p < 0.05$).

		TCP	LPS	Dex	Cu	Mg	CuD	MgD
24h	TCP		*	*	ns	*	*	*
	LPS			*	*	*	*	*
	Dex				*	*	*	ns
	Cu					*	*	*
	Mg						*	*
	CuD							*
	MgD							
48h	TCP		ns	*	*	*	*	*
	LPS			*	*	*	*	*
	Dex				*	*	*	ns
	Cu					ns	*	*
	Mg						*	*
	CuD							*
	MgD							

Table S7. Statistical analysis of the results of TNF-α expression when THP-1 macrophages were stimulated with a combination of Cu^{2+} or Mg^{2+} with dex in presence of the pro-inflammatory stimulus LPS. Statistically significant differences were indicated with *($p < 0.05$).

		TCP	LPS	Dex	Cu	Mg	CuD	MgD
24h	TCP		*	*	*	*	*	*
	LPS			*	*	ns	ns	*
	Dex				*	ns	*	ns
	Cu					ns	ns	ns
	Mg						ns	ns
	CuD							*
	MgD							
48h	TCP		*	*	*	*	*	*
	LPS			*	ns	ns	*	*
	Dex				*	ns	*	*
	Cu					ns	*	*
	Mg						ns	ns
	CuD							*
	MgD							

Table S8. Statistical analysis of the results of IL-1β expression when THP-1 macrophages were stimulated with a combination of Cu^{2+} or Mg^{2+} with dex in presence of the pro-inflammatory stimulus LPS. Statistically significant differences were indicated with *($p < 0.05$).

		TCP	LPS	Dex	Cu	Mg	CuD	MgD
24h	TCP		*	*	*	*	*	*
	LPS			*	*	ns	*	*
	Dex				*	ns	*	*
	Cu					*	*	ns
	Mg						*	*
	CuD							ns
	MgD							
48h	TCP		*	ns	*	ns	ns	ns
	LPS			*	*	ns	*	*
	Dex				ns	ns	ns	ns
	Cu					ns	ns	ns
	Mg						ns	ns
	CuD							ns
	MgD							

Table S9. Statistical analysis of the results of CCR7 expression when THP-1 macrophages were stimulated with a combination of Cu^{2+} or Mg^{2+} with dex in presence of the pro-inflammatory stimulus LPS. Statistically significant differences were indicated with *($p < 0.05$).

		TCP	LPS	Dex	Cu	Mg	CuD	MgD
24h	TCP		*	*	ns	*	*	*
	LPS			*	*	*	ns	ns
	Dex				ns	ns	ns	ns
	Cu					ns	ns	ns
	Mg						*	*
	CuD							ns
	MgD							
48h	TCP		ns	ns	ns	ns	ns	ns
	LPS			*	ns	*	ns	ns
	Dex				*	*	*	*
	Cu					ns	ns	ns
	Mg						ns	ns
	CuD							ns
	MgD							

Table S10. Statistical analysis of the results of IL-10 expression when THP-1 macrophages were stimulated with a combination of Cu^{2+} or Mg^{2+} with dex in presence of the pro-inflammatory stimulus LPS. Statistically significant differences were indicated with *($p < 0.05$).

		TCP	LPS	Dex	Cu	Mg	CuD	MgD
24h	TCP		*	*	ns	*	*	*
	LPS			*	ns	*	*	*
	Dex				ns	ns	*	*
	Cu					ns	ns	ns
	Mg						ns	ns
	CuD							ns
	MgD							
48h	TCP		*	ns	*	*	*	*
	LPS			*	ns	ns	*	*
	Dex				*	ns	ns	ns
	Cu					ns	ns	ns
	Mg						ns	ns
	CuD							ns
	MgD							

Table S11. Statistical analysis of the results of TGF-β expression when THP-1 macrophages were stimulated with a combination of Cu^{2+} or Mg^{2+} with dex in presence of the pro-inflammatory stimulus LPS. Statistically significant differences were indicated with *($p < 0.05$).

		TCP	LPS	Dex	Cu	Mg	CuD	MgD
24h	TCP		ns	*	ns	*	ns	ns
	LPS			*	ns	ns	ns	ns
	Dex				ns	ns	*	*
	Cu					ns	ns	ns
	Mg						*	*
	CuD							ns
	MgD							
48h	TCP		ns	ns	ns	ns	*	ns
	LPS			ns	ns	ns	*	ns
	Dex				ns	ns	*	ns
	Cu					ns	*	ns
	Mg						*	ns
	CuD							*
	MgD							

Table S12. Statistical analysis of the results of CD206 expression when THP-1 macrophages were stimulated with a combination of Cu^{2+} or Mg^{2+} with dex in presence of the pro-inflammatory stimulus LPS. Statistically significant differences were indicated with *($p < 0.05$).

		TCP	LPS	Dex	Cu	Mg	CuD	MgD
24h	TCP		*	*	ns	*	*	*
	LPS			*	*	*	*	*
	Dex				*	*	*	*
	Cu					*	*	*
	Mg						*	*
	CuD							*
	MgD							
48h	TCP		*	*	*	*	*	*
	LPS			*	ns	ns	*	*
	Dex				*	*	ns	ns
	Cu					ns	*	*
	Mg						*	*
	CuD							ns
	MgD							
